# Supplementary material for: Plumbagin, a Natural Product with Potent Anticancer Activities, Binds to and Inhibits Dihydroorotase, a Key Enzyme in Pyrimidine Biosynthesis
Source: Int J Mol Sci. 2021 Jun 25;22(13):6861. doi: 10.3390/ijms22136861 (PMC8267945; doi:10.3390/ijms22136861)
Supplement: Supplementary file 1 [file ijms-22-06861-s001.zip › ijms-1261400-supplementary.pdf]

SUPPLEMENTARY INFORMATION

Plumbagin, a natural product with potent anticancer activities, binds to and inhibits dihydroorotase, a key enzyme in pyrimidine biosynthesis

Hong-Hsiang Guan, Yen-Hua Huang, En-Shyh Lin, Chun-Jung Chen, and Cheng-Yang Huang

Supplementary information includes:

Figure S1: An alignment consensus of sequenced DHOase homologs by ConSurf. Figure S2: Binding of DDCA to ScDHOase. Figure S3: Binding of orotic acid to ScDHOase. Table S1: The formation of hydrogen bonds in the complexed structure of ScDHOase with PLU. Table S2: Primers used for construction of plasmids. Table S3: Anticancer activity of PLU and 5-FU against 4T1 cells.

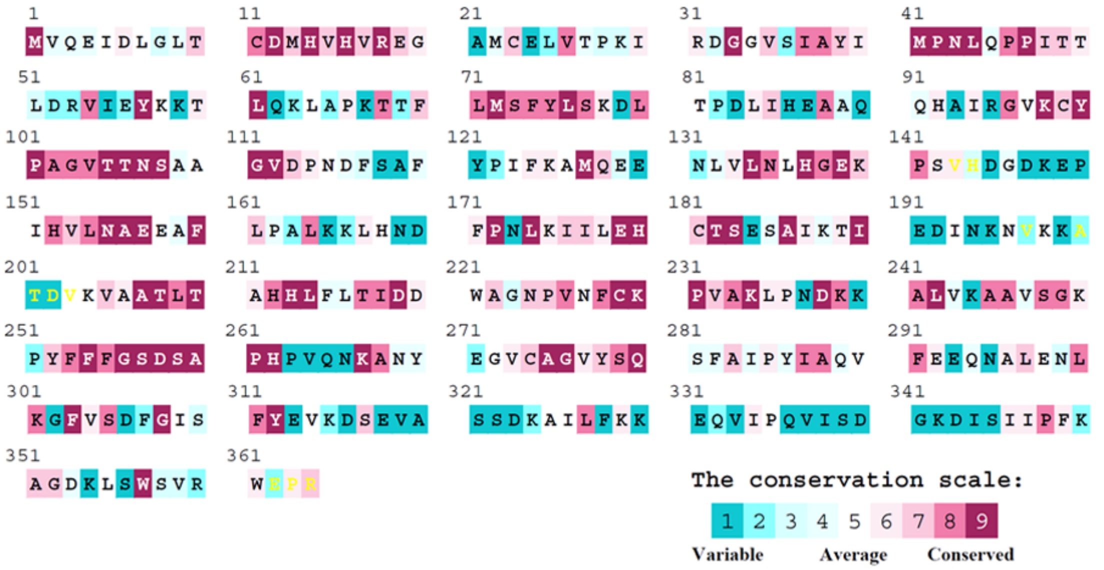

Supplementary Figure S1. An alignment consensus of sequenced DHOase homologs by ConSurf. To show the degree of variability at each position along the sequence, the alignment consensus of 1694 unique sequenced DHOase homologs was performed by ConSurf. Amino acid residues that are highly variable are colored teal, while highly conserved amino acid residues are colored burgundy. A consensus sequence was established by determining which amino acid residue is most commonly found at each position relative to the primary sequence of ScDHOase. The binding sites mentioned above are highly conserved among varying organisms.

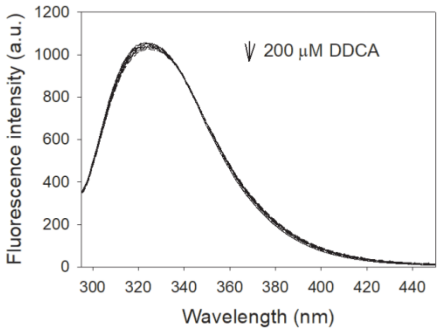

Supplementary Figure S2. Binding of DDCA to ScDHOase. The fluorescence emission spectra of ScDHOase with DDCA of different concentrations (0–200 μM). The decrease in intrinsic fluorescence of protein was measured at 324 nm upon excitation at 280 nm with a spectrofluorimeter. When DDCA (200 μM) was added into the ScDHOase solution, the intrinsic fluorescence was only quenched by 3.0%, suggesting low interactions.

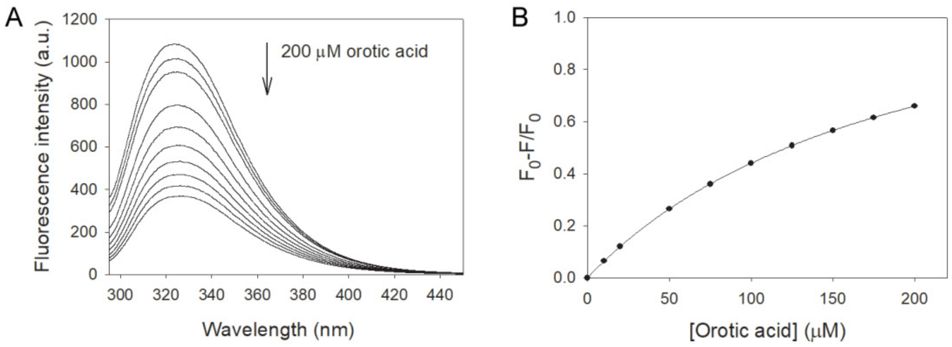

Supplementary Figure S3. Binding of orotic acid to ScDHOase. (A) The fluorescence emission spectra of ScDHOase with orotic acid of different concentrations (0–200 μM; 0, 10, 20, 50, 75, 100, 125, 150, 175, and 200 μM). The decrease in intrinsic fluorescence of protein was measured at 324 nm upon excitation at 280 nm with a spectrofluorimeter. The fluorescence intensity emission spectra of ScDHOase significantly quenched with orotic acid. (B) An aliquot amount of orotic acid was added to the enzyme solution for determining the K_d. The K_d was obtained by the equation: The K_d was obtained by the equation: $\Delta F = \Delta F_{max} - K_d(\Delta F/[\text{orotic acid}])$. Data points are an average of 2–3 determinations within 10% error. The K_d value of orotic acid for ScDHOase was 198.3 ± 1.2 μM as determined through this curve.

Supplementary Table S1. The formation of hydrogen bonds in the complexed structure of ScDHOase with PLU.

Subunit	Ligand	Distance (Å)	Residue
B	PLU [O2]	2.62	H16 [ND1]
B	PLU [O2]	3.11	R18 [NH1]
B	PLU [O2]	2.99	N43 [ND2]
B	PLU [O1]	2.01	K230 [O]
B	PLU [O3]	3.39	K230 [N]
D	PLU [O2]	2.97	T105 [OG1]
D	PLU [O1]	3.59	D258 [O]
D	PLU [O1]	2.54	D258 [OD2]
A	Malate [O1B]	2.83	R18 [NH2]
A	Malate [O2]	3.66	N43 [ND2]
A	Malate [O1A]	2.35	H262 [NE2]
C	Malate [O4A]	2.24	R18 [NH1]
C	Malate [O4A]	3.75	N43 [ND2]
C	Malate [O2]	2.34	T105 [OG1]
C	Malate [O2]	3.44	T106 [N]
C	Malate [O2]	3.77	T106 [O]
C	Malate [O4B]	3.21	T106 [OG1]

The formation of hydrogen bonds was analyzed by PISA, an automatic analytical tool for macromolecular assemblies in the crystalline state.

Supplementary Table S2. Primers used for construction of plasmids.

Oligonucleotide	Primer
ScDHOase-wt-NdeI-N	GGGCATATGGTACAAGAAATCGATTTA
ScDHOase-wt-XhoI-C	GGGCTCGAGACGAGGTTCCCATCTCAC
ScDHOase H14A-N	TTGACATGTGATATGGCTGTTCATGTAAGA
ScDHOase H14A-C	CTCTCTTACATGAACAGCCATATCACATGT
ScDHOase H16A-N	ACATGTGATATGCATGTTGCTGTAAGAGAG
ScDHOase H16A-C	CGCACCCTCTCTTACAGCAACATGCATATC
ScDHOase R18A-N	GCATGTTCATGTAGCAGAGGGTGCGATGTG
ScDHOase R18A-C	TCGCACCCTCTGCTACATGAACATGCATAT
ScDHOase N43A-N	TACATCATGCCAGCTTTACAACCTCCAATT
ScDHOase N43A-C	TGGAGGTTGTAAAGCTGGCATGATGTAAGC
ScDHOase K98A-N	TGCCATTCGTGGAGTAGCGTGTTATCCAGC
ScDHOase K98A-C	TCCCGCTGGATAACACGCTACTCCACGAAT

ScDHOase T105A-N	CCAGCGGGAGTAGCAACAAATTCGGCTGCT
ScDHOase T105A-C	CCGAATTTGTTGCTACTCCCGCTGGATAAC
ScDHOase T106A-N	CCAGCGGGAGTAACAGCAAATTCGGCTGCT
ScDHOase T106A-C	CCGAATTTGCTGTTACTCCCGCTGGATAAC
ScDHOase H137A-N	ACCTGGTATTAAATTTGGCTGGGGAAAAAC
ScDHOase H137A-C	CAGAAGGTTTTTCCCCAGCCAAATTTAATA
ScDHOase H180A-N	TAATTCTGGAAGCCTGCACTAGCGAGTCGG
ScDHOase H180A-C	CGCTAGTGCAGGCTTCCAGAATTATTTTCA
ScDHOase D258A-N	CTTTGGATCTGCTTCAGCACCTCATCCTG
ScDHOase D258A-C	CAGGATGAGGTGCTGAAGCAGATCCAAAG
ScDHOase D258E-N	TCTTTGGATCTGAATCAGCACCTCATCCTG
ScDHOase D258E-C	CAGGATGAGGTGCTGATTCAGATCCAAAGA
ScDHOase H262A-N	ATTCAGCACCTGCTCCTGTACAAAATAAGG
ScDHOase H262A-C	TTGTACAGGAGCAGGTGCTGAATCAGATC
huDHOase1456-1846-NdeI-N	GGGCATATGACCTCCCAAAAGCTTGTG
huDHOase1456-1846-XhoI-C	GGGCTCGAGATCAGGAAGCCCTGGGATGCC
CAD(aa1612 TAT to TCT)-N	GCTGAGTGCACATCTGTCACGTGGCAC
CAD(aa1612 TAT to TCT)-C	GTGCCACGTGACAGATGTGCACTCAGC
StDHOase-wt-NdeI-N	AAGATCATATGACTGCACCATCCCAGG
StDHOase-wt-XhoI-C	ACTGGCTCGAGTTTTTTTACTGACCAG

These plasmids were verified by DNA sequencing. Underlined nucleotides indicate the designated site for mutation or the restriction site.

Supplementary Table S3. Anticancer activity of PLU and 5-FU against 4T1 cells.

Cytotoxicity	None	PLU (2 μM)	5-FU (5 μM)	PLU (2 μM) and 5-FU (5 μM)
Death rate (%)	0	8	27	59
Migration rate (%)	99	84	65	37
Apoptotic rate (%)	1	8	26	58
Colony count (%)	100	73	54	17

Value is an average of 3 determinations within 10% error.
